# Plasma bioactive adrenomedullin on intensive care unit admission is associated with acute respiratory distress syndrome: an observational study

**DOI:** 10.1186/s40635-023-00494-7

**Published:** 2023-03-03

**Authors:** Patrik Johnsson, Andrea Fredriksson, Christer Tung, Hans Friberg, Attila Frigyesi

**Affiliations:** 1 Department of Clinical Medicine, Anaesthesiology and Intensive Care, Lund University, 22185 Lund, Sweden; 2 Department of Intensive and Perioperative Care, Skåne University Hospital, 20502 Malmo, Sweden

**Keywords:** Adult respiratory distress syndrome, Biomarker, Adrenomedullin, Endothelial function, COVID-19, Sepsis

## Abstract

**Background:**

Bioactive adrenomedullin (bio-ADM) is a vasoactive peptide with a key role in reducing vascular hyperpermeability and improving endothelial stability during infection, but it also has vasodilatory properties. Bioactive ADM has not been studied in conjunction with acute respiratory distress syndrome (ARDS), but it has recently been shown to correlate with outcomes after severe COVID-19. Therefore, this study investigated the association between circulating bio-ADM on intensive care unit (ICU) admission and ARDS. The secondary aim was the association between bio-ADM and ARDS mortality.

**Methods:**

We analysed bio-ADM levels and assessed the presence of ARDS in adult patients admitted to two general intensive care units in southern Sweden. Medical records were manually screened for the ARDS Berlin criteria. The association between bio-ADM levels and ARDS and mortality in ARDS patients was analysed using logistic regression and receiver-operating characteristics analysis. The primary outcome was an ARDS diagnosis within 72 h of ICU admission, and the secondary outcome was 30-day mortality.

**Results:**

Out of 1224 admissions, 11% (*n* = 132) developed ARDS within 72 h. We found that elevated admission bio-ADM level was associated with ARDS independently of sepsis status and of organ dysfunction as measured by the Sequential organ failure assessment (SOFA) score. Both *lower* levels (< 38 pg/L) and high (> 90 pg/L) levels of bio-ADM were independently (of the Simplified acute physiology score, SAPS-3) predictive of mortality. Patients with indirect mechanisms of lung injury had higher bio-ADM levels than those with a direct mechanism of injury, and bio-ADM increased with increasing ARDS severity.

**Conclusions:**

High levels of bio-ADM on admission are associated with ARDS, and bio-ADM levels significantly differ depending on the injury mechanism. In contrast, both high and low levels of bio-ADM are associated with mortality, possibly due to the dual action of bio-ADM in stabilising the endothelial barrier and causing vasodilation. These findings could lead to improved diagnostic accuracy of ARDS and potentially lead to new therapeutic strategies.

**Supplementary Information:**

The online version contains supplementary material available at 10.1186/s40635-023-00494-7.

## Introduction

### ARDS

Acute respiratory distress syndrome (ARDS) is a clinical diagnosis associated with hypoxic respiratory failure often caused by pulmonary endothelial damage and inflammation. Since first described by Ashbaugh et al. in 1967 [1], the hallmarks of this disorder have not changed. It is characterised by a rapid onset and high mortality [2, 3]. The resulting hypoxemia results from injury to the lung tissue through either direct or indirect mechanisms. Damaged pulmonary vessel endothelial and alveolar epithelial cells cause alveolar and interstitial oedema preventing adequate gas exchange [4], in the exudative phase of ARDS. The damaged alveolar epithelium’s compromised integrity and barrier function is restored by fibroblasts that deposit fibronectin and collagen and form an interstitial extracellular matrix in the proliferative phase. In patients where the epithelial integrity fails to be restored, marked deposition of extracellular matrix can lead to pulmonary fibrosis [5]. Due to the multifaceted clinical presentation of ARDS risk factors the diagnosis is, in many instances, unrecognised by clinicians.

For example, in a large international multicentre prospective cohort study in the intensive care unit (ICU) published in 2016 only 60% of ARDS patients were properly recognised, including those with severe disease, and the diagnosis was frequently delayed with only 34% of cases recognised at the time of ARDS criteria fulfilment [2]. Delayed diagnosis or missed cases of ARDS are unfortunate as there are simple interventions proven to reduce mortality, such as prone positioning, neuromuscular blockers, and lung-protective ventilation [6].

Biomarkers and their role in ARDS diagnosis, prognostication, identification of phenotypes, and potential targets for treatment have been studied for decades. Through the use of biomarkers or clinical data, various ARDS phenotypes have been identified, such as hypo-inflammatory versus hyper-inflammatory phenotypes or direct (epithelial) lung injury vs indirect (endothelial) lung injury [7]. Furthermore, these phenotypes seem to respond differently to therapeutic interventions such as high positive end-expiratory pressure (PEEP) levels, fluid therapy, and statins [7, 8]. Unfortunately, the classification of ARDS based on biomarkers or other clinical characteristics to facilitate more targeted interventions has not been adequately investigated yet. Diagnosis is still based on the clinical Berlin criteria [3], and the available treatment is supportive only.

### Adrenomedullin

Bioactive adrenomedullin (bio-ADM) is a vasoactive hormone with multiple roles in homeostasis and was first isolated from human pheochromocytoma [9]. It is found in many tissues throughout the body, such as cardiac, renal, and vascular tissue. It is also present in high concentration in the lungs [10, 11]. Bio-ADM is also extensively expressed in endothelial cells, where it can induce vasodilation and stabilise the endothelial barrier preventing vascular leakage [12–15]. In addition, bio-ADM moves freely between the blood and into the interstitial compartment, where it relaxes vascular smooth muscle cells [12].

Bio-ADM has been extensively studied in sepsis, and high levels of ADM correlate with disease severity and mortality, [16–18]. Treatment with bio-ADM or the antibody adrecizumab, which increases functional ADM in plasma, has been suggested as a future treatment of sepsis [19]. Adrecizumab was successfully evaluated for safety and tolerability in a recent phase-2a trial in patients with sepsis and elevated bio-ADM. The rationale is that adrecizumab binds and increases plasma ADM, thereby promoting vascular integrity while simultaneously attenuating vasodilation through decreased ADM concentrations in the interstitium [12, 20]. Adrenomedullin has also shown promise as a treatment to prevent or attenuate lung injury in experimentally induced sepsis and ventilator-induced lung injury (VILI) by strengthening the endothelial barrier and reducing alveolar oedema [21, 22]. Recently, several studies have shown a relationship between high levels of adrenomedullin and severity of illness and mortality in hospitalised COVID-19 patients [23, 24], and adrecizumab has been tested as a treatment for severe COVID-19 ARDS in a small uncontrolled case series [25].

To our knowledge, bio-ADM has not been evaluated as a diagnostic and prognostic biomarker in ARDS in a general ICU population.

### Objectives

The primary aim of this study was to investigate the association between bio-ADM levels and ARDS in ICU patients. The secondary aim was to investigate the association of ICU admission bio-ADM with 30-day mortality in ARDS patients.

## Methods

### Study design

The Strengthening the Reporting of Observational Studies in Epidemiology (STROBE) guidelines were followed [26].

We retrospectively collected clinical data on patients admitted to the intensive care unit (ICU) at two hospitals in southern Sweden in 2016 to identify patients with ARDS and their controls, non-ARDS. Using prospectively collected ICU admission blood samples, we compared ADM levels between ARDS patients and controls to find an association between ADM levels on admission and the prevalence of ARDS. Blood samples were collected and stored in a bio-bank. Next of kin were informed of admission and could opt out on the patient’s behalf. All intensive care survivors were sent an information letter two months after discharge and given the possibility of opting out. If the patient or next of kin opted out, the samples were destroyed and the patient was excluded from the study.

### Setting

Adult patients admitted to two general mixed ICUs at Skåne University Hospital in Lund and Malmö in southern Sweden between January 1st 2016, and December 31st 2016, were considered eligible for the study. Clinical data for the entire duration of their ICU stay were retrospectively collected, and data on mortality were collected. In addition, blood samples were prospectively collected on ICU admission and stored in a bio-bank.

### Participants

Using the Patient Administrative System for Intensive Care Units (PASIVA), we identified all patients admitted to the participating ICUs during the study period. Adult patients (18 years or older) were eligible if they had valid blood samples and had not opted out of the study. Patients who fulfilled the Berlin criteria for ARDS within 72 h of ICU admission were considered cases. The patients who did not fulfil the criteria were allocated to a control group.

### Variables

The primary outcome was ARDS prevalence. The secondary outcome was 30-day mortality in patients fulfilling the ARDS criteria.

The Berlin definition was used to identify and define ARDS patients, but with a modification of the timing criteria (see below) [3]:

- Debut within 1 week of a known clinical insult or new or worsening respiratory symptoms
- Bilateral opacities on chest radiograph or computed tomography not explained by effusions, lobar/lung collapse, or nodules
- Respiratory failure not fully explained by cardiac failure or fluid overload
- P$_\text {a}$O$_{2}$/F$_\text {i}$O$_{2}$ $\le$ 40 kPa with PEEP or CPAP $\ge$ 5 cm H$_{2}$O

Instead of the seven-day timing window of the original definition, only patients who developed ARDS within 72 h were included as cases. Clinical data were collected by trained data collectors who did not have access to radiology or biochemistry. A sub-specialised cardio-thoracic radiologist assessed chest imaging, blinded to the clinical data. If the patient fulfilled all the criteria within the same 24-h period and had a risk factor present on ICU admission, they were considered a case. The onset of ARDS was defined as the time when the patient first fulfilled all criteria. ARDS severity was based on the worst daily mean P$\text {a}$O$_{2}$/F$_{i}$O$_{2}$ from the day of ARDS debut and up to 24 h afterwards. Means were trimmed by excluding 20% of the lowest and highest values.

Sepsis of all causes was defined according to the 2016 Sepsis-3 criteria [27].

Comorbidities were classified according to the updated Charlson’s Comorbidity Index [28], the Simplified Acute Physiology Score 3 (SAPS-3) [29] and the Sequential Organ Failure Assessment (SOFA) score [30]. Both SAPS-3 and SOFA were calculated within one hour of admission.

Bioactive adrenomedullin (bio-ADM) was analysed from plasma samples collected and frozen within 6 h of ICU admission.

### Data sources/measurement

Clinical data on risk factors, comorbidities, and treatment variables from electronic medical records were retrospectively collected by trained data collectors using a list of predefined variables. Uncertainties in classification were decided collectively in the study group. Biochemical results were automatically extracted from the patient’s electronic medical records.

The collection of data for the identification of Sepsis-3 patients has been described in detail elsewhere [31].

The SOFA score, the SAPS-3, and survival data were extracted from PASIVA, where the treating physicians and nurses prospectively entered physiologic and treatment data. Survival data were downloaded from the national population registry.

Chest radiographs and chest CTs were evaluated using the radiologic criteria of the Berlin definition. The first 100 reviewed images were compared to the example images of the Berlin definition supplementary material and reviewed in the study group with a second cardio-thoracic radiologist to ensure that the criteria were correctly applied. The radiologist was blinded to the clinical and laboratory data, and imaging findings were assessed as ’consistent with ARDS’, ’equivocal’, or ’inconsistent with ARDS’. Studies assessed as ’consistent with ARDS’ were considered diagnostic.

Blood samples were collected in EDTA vacutainers, centrifuged to obtain plasma, aliquoted, and frozen. Before analysis, frozen plasma samples were stored in the SWECRIT biobank at Region Scania (BD-47, SC-1922). Samples were shipped, and batch analysis of bio-ADM was performed on thawed samples in 2019 at the laboratory of SphingoTec GmbH (Hennigsdorf, Germany). The assay has previously been described elsewhere [32]

### Bias

All manual data collection was performed in a systematic way using predefined variables. Uncertainties were decided on collectively in the study group. Baseline characteristics between included and excluded patients were compared to rule out selection bias. Since a lack of data could lead to the misclassification of ARDS patients, we examined the number of patients who did not fulfil the ARDS criteria due to missing data.

### Study size

The study size was a convenience sample of the number of ICU patients with valid blood samples during the study period.

### Statistical analysis

For all hypothesis tests, we considered *p*-values < 0.05 as significant. To assess a difference in the location of two independent variables, we used the Wilcoxon rank-sum test (Mann–Whitney *U* test). Differences in proportions were assessed using Pearson’s $\chi ^2$ test. Medians were reported with corresponding interquartile ranges (IQR), while the mean was reported with its standard deviation (SD). Logistic regression was used to analyse outcomes. The results of the regression analyses are reported as odds ratios (OR) with 95% confidence intervals (CI). The Bio-ADM levels were transformed with the natural logarithm due to skewness in the regression analyses. Areas under the curve (AUC) were derived from the receiver operating characteristic (ROC) curves [33]. Differences in AUCs were assessed with the method of DeLong et al. [34]. Admissions with missing data for any variable were excluded from mean and median calculations. In addition, the number of missing observations was specified. To adjust for the severity of the disease, the non-respiratory elements of the SOFA score were included in the regression model.

## Results

### Participants

**Fig. 1:**
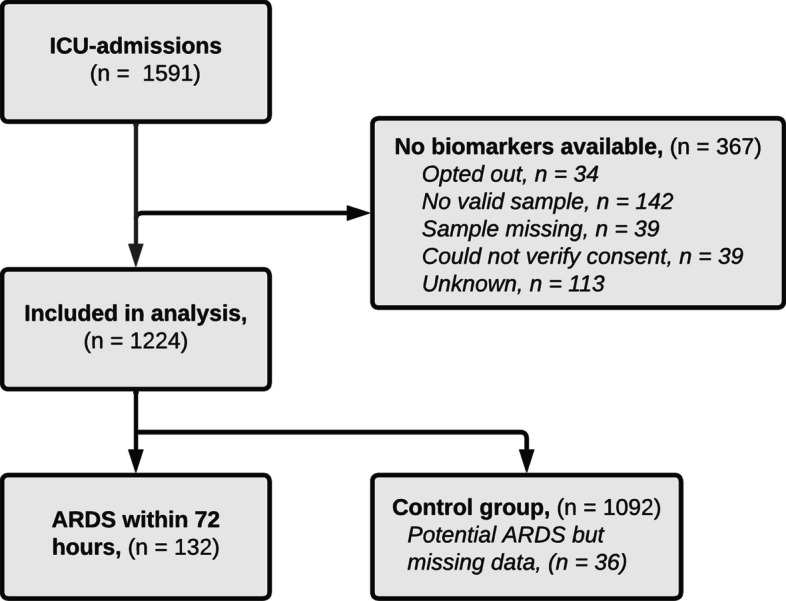
Flowchart of the study. *ICU* intensive care unit, *ARDS* acute respiratory distress syndrome

A total of 1591 patients were admitted to the participating ICUs during the study period. Of these, 367 patients had no available bio-markers due to missing blood samples or lack of consent. In total, 1224 patients were included in the study. See the flowchart in Fig. 1.

### Demographics/descriptive data

The ARDS criteria were fulfilled in 132 of 1224 patients (10%) within 72 h of ICU admission. Of the 1092 patients in the control group, 36 fulfilled three out of four ARDS criteria within 72 h but had no chest imaging available (see Additional file 1: Table S1). Of the patients without a known ARDS risk factor, only a minority (43%) had an echocardiographic examination, and none fulfilled the ARDS criteria. The median time from ICU admission to ARDS diagnosis was 4 h [1.0–9.0].

**Table 1:** Characteristics of ARDS-patients and controls. Data regarding general characteristics, outcomes, organ dysfunction and illness severity are presented below

Parameter	ARDS	Control	*p*-value	Missing (%)
Demographics				
*n*	132	1092		
Female, *n* (%)	43 (33)	429 (39)	0.2	
Age, years, mean (SD)	64 (14)	62 (17)	0.1	
BMI, kg/m^2^, mean (SD)	26.5 (6.3)	26.9 (6.7)	0.6	30
Comorbidities, *n* (%)				
CCI score, mean (SD)	3.6 (2.4)	3.6 (2.8)	1.0	
Cardiac arrest, *n* (%)	4 (3.0)	11 (1.0)	0.1	
Sepsis-3 criteria, *n* (%)	89 (67.4)	317 (29.0)	< 0.001	
Direct lung-injury risk factors, *n* (%)				
Pneumonia	95 (72)	132 (12)	< 0.001	
Aspiration	32 (24)	71 (7)	< 0.001	
Inhalational injury	0 (0.0)	3 (0.3)	1.0	
Drowning	0 (0.0)	2 (0.2)	1.0	
Lung contusion	4 (3.0)	23 (2.1)	0.7	
Pulmonary vasculitis	4 (3.0)	3 (0.3)	< 0.001	
Other	2 (1.5)	1 (0.1)	0.03	
None	15 (11)	887 (81)	< 0.001	
Indirect lung-injury risk factors, *n* (%)				
Extrapulmonary sepsis	38 (28.8)	163 (14.9)	< 0.001	
Severe burns	0 (0.0)	1 (0.1)	1.0	
Non-cardiogenic shock	7 (5.3)	92 (8.4)	0.3	
Drug overdose	7 (5.3)	59 (5.4)	1.0	
Multiple blood transfusions	9 (6.8)	106 (9.7)	0.4	
Trauma	2 (1.5)	94 (8.6)	0.007	
Pancreatitis	1 (0.8)	10 (0.9)	1.0	
Other	0 (0)	30 (2.7)	0.1	
None	76 (57.6)	645 (59.1)	0.8	
Severity of illness, mean (SD)				
SAPS 3 score	72.3 (14.0)	57.5 (16.1)	< 0.001	
SOFA score	9.8 (3.8)	6.0 (3.6)	< 0.001	12
Respiratory SOFA	3.3 (0.7)	1.9 (1.3)	< 0.001	19
Coagulation SOFA	0.5 (0.9)	0.3 (0.7)	< 0.001	10
Neurologic SOFA	1.6 (1.5)	1.3 (1.5)	0.007	2
Renal SOFA	1.4 (1.5)	0.8 (1.3)	< 0.001	10
Circulatory SOFA	2.5 (1.6)	1.7 (1.6)	< 0.001	2
Hepatic SOFA	0.4 (0.9)	0.3 (0.6)	0.009	12
Biochemistry				
ADM, pg/L, median [IQR]	65.6 [38.4, 125.5]	35.4 [20.2, 71.4]	< 0.001	
CRP, mg/L, median [IQR]	99 [33, 230]	21 [4, 98]	< 0.001	1.8
Leukocytes, 10^9^/L, median [IQR]	14 [9, 19]	13 [9, 18]	0.6	1.0
Lactate, mmol/L, median [IQR]	3.1 [1.8, 5.2]	2.3 [1.4, 4.4]	0.003	4.9
Creatinine, µmol/L, median [IQR]	120 [84, 189]	93 [71, 135]	< 0.001	1.1
ARDS severity, *n* (%)				
Mild ARDS	27 (21)	–		
Moderate ARDS	81 (61)	–		
Severe ARDS	27 (21)	–		
Outcomes				
30-day mortality, *n* (%)	52 (39)	194 (18)	< 0.001	
Duration of ICU stay, d, median [IQR]	3.5 [1.7, 6.5]	1.4 [0.7, 2.9]	< 0.001	
Duration of mechanical ventilation, d, median [IQR]	2.3 [1.1, 5.3]	1.1 [0.4, 2.7]	< 0.001	

ARDS patients were compared to controls, and the *p*-values refer to that comparison. Proportions (%) are within their subgroups unless otherwise specified. SD: standard deviation; IQR: interquartile range; CCI: Charlson Comorbidity Index; SAPS3: Simplified Acute Physiology Score III; SOFA: Sequential Organ Failure Assessment; bio-ADM: circulating bioactive adrenomedullin; CRP: C-Reactive Protein; SOFA: sequential organ failure assessment; ICU: intensive care unit

Baseline characteristics are presented in Table 1. Age, sex, BMI, and comorbidities according to the Charlson Comorbidity Index did not differ significantly between ARDS patients and controls (Table 1). Pulmonary risk factors were more common in the ARDS group, particularly pneumonia and aspiration. Non-pulmonary risk factors such as sepsis and septic shock were more common in the ARDS group, while trauma was more common in the control group. Sepsis of all causes (sepsis-3) was significantly more common in ARDS patients (67.4% vs 29.0%, *p *< 0.001) Thirty-day mortality was higher in ARDS patients (39.4% vs 17.9%, *p *< 0.001 in the control group), as were the duration of ICU stay and mechanical ventilation. On admission, both mean SOFA scores (9.75 vs 6.0, *p *< 0.001), mean non-pulmonary SOFA scores, and SAPS-3 scores were significantly higher in the ARDS group. A single extreme value was found for bio-ADM (4568 pg/L), which was excluded from the analysis. Bio-ADM values ranged from 8 pg/L to 1819 pg/L. The median bio-ADM levels were higher in ARDS patients (62.7 pg/L vs 35.6 pg/L for controls, *p *< 0.001).

**Fig. 2:**
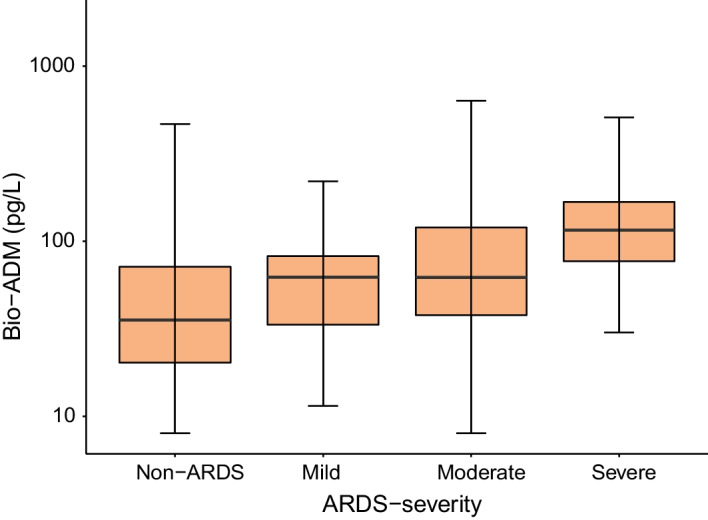
Bio-ADM vs ARDS severity. Bio-ADM: circulating bioactive adrenomedullin, ARDS: acute respiratory distress syndrome

Demographics and comorbidities did not differ with ARDS severity. Mortality was the highest in patients with severe ARDS (56%) compared to 40% and 15% for moderate and mild ARDS, respectively (*p* = 0.040). The median number of days on mechanical ventilation increased from 1.8 [1.1–4.5] days for mild ARDS, 2.1 [1.0–5.1] days for moderate ARDS, to 4.17 [1.24–7.9] days for severe ARDS, where the difference between mild to moderate and severe ARDS was significant (*p* = 0.023). The SOFA score and the SAPS-3 increased with the severity of ARDS. Bio-ADM values were higher in severe ARDS than in mild or moderate ARDS (median values 115.3, 62.1 and 62.3 pg/L, respectively, *p* = 0.0070), see Fig. 2.

Compared to those included in the study, excluded patients had a slightly lower Charlson comorbidity index (3.1 vs 3.6, *p *<  0.001), and cardiac arrest was more common (3.5% vs 1.2%, *p* = 0.002). Fewer had ARDS (5.7% vs 10.8%, *p* = 0.005) and mortality was higher (32% vs 20%, *p *< 0.001, see Additional file 1: Table S2).

### Bio-ADM and ARDS development

**Fig. 3:**
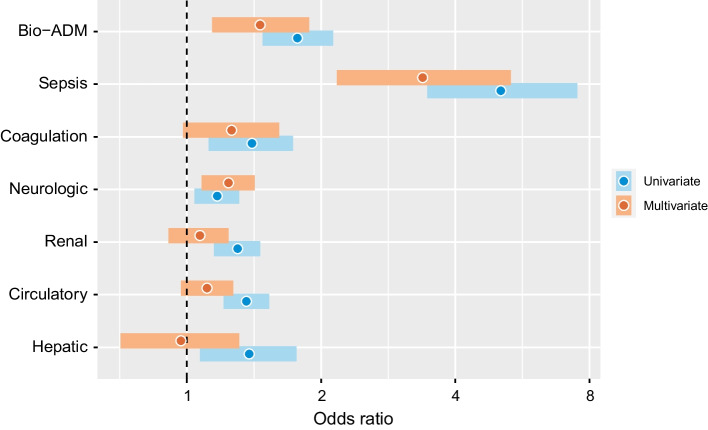
The association between ARDS, bio-ADM levels, and organ dysfunction as measured by SOFA score on admission with odds ratios (log-2 scale) and 95% confidence intervals from univariate and multivariate logistic regression. Bio-ADM: circulating bioactive adrenomedullin, ARDS: cute respiratory distress syndrome. SOFA: sequential organ failure assessment

In univariate logistic regression analyses, bio-ADM (OR 1.8, 95% CI 1.5–2.1, *p* < 0.001), Sepsis-3 criteria (OR 5.1, 95% CI 3.5–7.5, *p* < 0.001) and all five types of non-respiratory organ dysfunction measured by SOFA were associated with ARDS. See Fig. 3.

In a multivariable logistic regression analysis of ARDS with bio-ADM, sepsis, and the non-respiratory SOFA scores as independent variables, bio-ADM was independently associated with ARDS (OR 1.5, 95% CI 1.1–1.9, *p* = 0.0030), as was sepsis (OR 3.4 95 % CI 2.2–5.3, *p* < 0.001. In addition, neurological SOFA (OR 1.2, 95% CI 1.1–1.4, *p* < 0.001) was also associated with ARDS.

Furthermore, in a ROC analysis bio-ADM had an AUC of 0.69 (95% CI 0.63–0.74) for the presence of ARDS.

The non-respiratory SOFA scores and Sepsis-3 had an AUC of 0.76 (95% CI 0.71–0.80), and the addition of ADM yielded an AUC of 0.77 (95% CI 0.72–0.75, *p* = 0.21).

### Bio-ADM and ARDS mechanism

The median bio-ADM value for ARDS solely caused by direct (pulmonary) risk factors was 66 [38–87,125] pg/L and 84 [75–180] pg/L for ARDS patients with any indirect risk factor of ARDS (*p* = 0.0023). Both groups had median bio-ADM values higher than that of the control group.

### Bio-ADM and mortality in ARDS

**Fig. 4:**
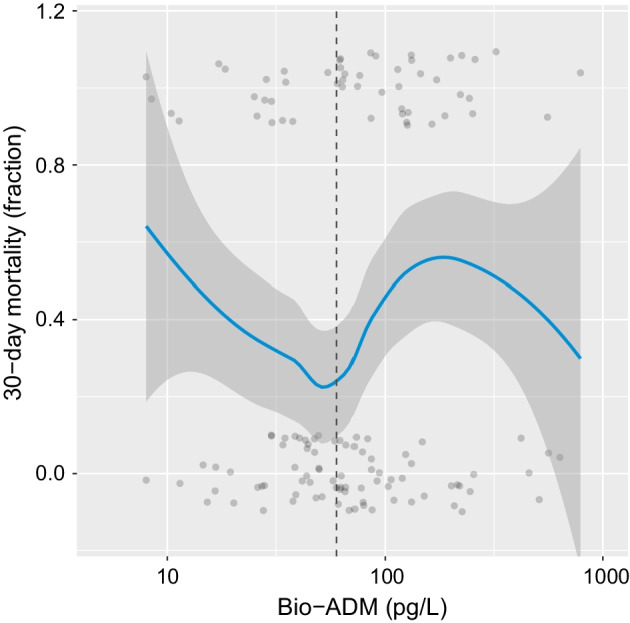
Thirty-day mortality vs bio-ADM for ARDS patients. Bio-ADM circulating bioactive adrenomedullin, ARDS acute respiratory distress syndrome. The dotted vertical line marks bio-ADM 60 pg/L

Thirty-day mortality vs admission bio-ADM levels are presented in Fig. 4. In a multivariate logistic regression analysis of mortality in ARDS with bio-ADM and disease severity as measured by SAPS-3 as independent variables, both *low* levels (< 38 pg/L) and *high* (> 90 pg/L) levels of bio-ADM were positively associated with mortality (OR$_\text {low}$ 3.39, 95% CI 1.17–10.36, *p* = 0.027 and OR$_\text {high}$ 3.08, 95% CI 1.06–9.25, *p* = 0.040).

## Discussion

In this study of intensive care patients, we used a rigorous and structured approach to manually identify all ARDS patients in the cohort without relying on diagnoses set by the treating clinician, as this is unrealiable [2]. We found that high admission levels of the vasoactive peptide bio-ADM were associated with fulfilling ARDS criteria within 72 h independently of the severity of the disease as measured by SOFA and independently of sepsis status. We also found that median bio-ADM levels increased with ARDS severity. Furthermore, ARDS patients with indirect mechanisms of lung injury had higher median values than those with a direct mechanism of lung injury. A U-shaped relationship between bio-ADM and ARDS mortality was seen, with the lowest mortality around 60 pg/L.

As expected from the properties of bio-ADM and a study in COVID-19 patients [23], we found that increasing levels of bio-ADM were positively associated with ARDS severity. Higher levels of bio-ADM were also strongly associated with fulfilling the ARDS criteria on ICU admission or soon thereafter. This association was independent of sepsis status according to Sepsis-3 criteria, meaning that high bio-ADM is not merely a marker of sepsis among ARDS patients. Bio-ADM does not just correlate with the degree of morbidity but could be associated with the mechanism of ARDS development. Endothelial injury is a hallmark of the exudative phase of ARDS development, so elevated levels of bio-ADM may reflect endothelial injury and a physiological compensatory mechanism due to increasing alveolar oedema.

Bio-ADM levels were higher in indirect lung injury mechanisms compared to direct lung injury. Considering that sampling was done within hours of ICU admission, and since bio-ADM is primarily produced in endothelial cells, this difference makes sense from a pathophysiological perspective. Direct mechanisms of lung injury initially have a more severe epithelial injury, while indirect mechanisms primarily have an endothelial or vascular injury in the early stages of the disease [35]. Indeed, these two clinical phenotypes (direct and indirect lung injury) show distinct patterns of biomarkers of epithelial and endothelial injury [36]. As the disease progresses, both epithelial cells and endothelial cells are eventually affected, as shown in a study of patients with severe non-pulmonary sepsis in the ICU, where epithelial biomarkers taken on the second day of intensive care can distinguish those who develop ARDS from those who do not, possibly indicating a progression from endothelial to epithelial injury [37].

The U-shaped relationship between bio-ADM and ARDS mortality was an unexpected finding. However, the present study was not designed to investigate this non-linear relationship. Mortality was high for *low levels* (defined as < 38 pg/L) and high levels (> 90 pg/L). Conversely, the lowest mortality in ARDS was seen in bio-ADM levels, around 60 pg/L. As bio-ADM is known to have a stabilising effect on the endothelium, the connection between *mortality* in ARDS and low levels of bio-ADM could be due to inadequate ADM production leading to endothelial dysfunction and vascular leakage in the context of widespread endothelial injury. The link between high levels of adrenomedullin and mortality has previously been shown for sepsis and may be due to marked vasodilation and hypotension, as described earlier. In various studies of sepsis and bio-ADM, cut-offs of 70, 102 and 108 pg/L have been shown to distinguish survivors from non-survivors [16–18]. However, in these studies, the relationship between bio-ADM and mortality was assumed to be linear. The non-linear relationship between bio-ADM levels and ARDS mortality should be further studied.

Interestingly, apart from the obvious connection between respiratory SOFA and ARDS, neurological SOFA was the organ failure most correlated with ARDS in multivariate regression models. Whether this is due to affected lungs or an increased risk of aspiration in patients with an altered level of consciousness, or if it is caused by brain-lung organ cross-talk [38], is beyond the scope of this study.

There were several limitations to our study. First, clinical data were collected retrospectively, and thus missing data could lead to the misclassification of patients. In particular, the limiting factors are missing chest imaging in patients who fulfil the other ARDS criteria and the lack of echocardiography in patients without a known risk factor. However, since the ARDS criteria were rigorously applied, we assess that this would primarily lead to erroneously classifying ARDS patients as controls, thus introducing bias towards the null hypothesis.

Among excluded patients, ARDS was less common. Since mortality was also significantly higher in excluded patients, it is possible that some of these patients were so critically ill on arrival that there was not enough time or resources to take the study samples or perform the necessary investigations needed to screen for ARDS.

Since bio-ADM was analysed within 6 h of ICU admission and many patients had or developed ARDS within the same time frame, this study cannot determine if high bio-ADM levels can predict ARDS development. It can only show a strong association between high bio-ADM and the presence of ARDS in critically ill patients.

The rationale for the deviation from the timing criteria of the Berlin definition (onset within 7 days) was that the majority of patients develop ARDS within 72 h [2, 3] and that ADM samples within hours of entry to the ICU would not be representative of the patient’s physiology after a week of intensive care.

Respiratory SOFA was excluded from the regression model since it is a part of the outcome of interest, i.e. respiratory SOFA is determined by the P$_\text {a}$O$_{2}$/F$_\text {i}$O$_{2}$-quotient, which is an intrinsic part of the definition of ARDS (P$_\text {a}$O$_{2}$/F$_\text {i}$O$_{2}$ $\le$ 40 kPa). Using non-respiratory SOFA allowed us to adjust for the severity of the disease without adjusting for the degree of respiratory failure.

The non-linear association between admission bio-ADM and mortality was interesting but had weak prognostic significance for 30-day mortality. This is unsurprising since bio-ADM was analysed at the start of intensive care, sometimes weeks before the patient died. In addition, the cut-offs for high and low bio-ADM in ARDS mortality were decided entirely ad-hoc, and further studies are needed to establish these levels.

Serial blood samples would be helpful in further elucidating the role of bio-ADM in ARDS pathophysiology and mortality. Our current samples are limited to ICU admission only.

The findings of this study have potential implications for a better understanding of the pathophysiology of ARDS and for managing ARDS patients in a general ICU population and possibly other critically ill patients with conditions characterised by endothelial injury and elevated levels of bio-ADM. If bio-ADM at a later date is shown to be predictive of ARDS development, it could be integrated into standard critical care since it is currently available as a bedside analysis. However, additional research is needed before this is feasible, especially serial sampling of bio-ADM and sampling before ICU admission (e.g. in the emergency department or general ward) to elucidate further the role of adrenomedullin in the pathogenesis of ARDS and to properly evaluate bio-ADM as a predictor of ARDS development and mortality. The safety and efficacy of treatment with adrenomedullin or adrecizumab in sepsis and ARDS also need further confirmation. Furthermore, serial studies in both classic ARDS and severe COVID-19 would help to further determine the role of ADM in the pathophysiology of severe respiratory disease.

## Conclusion

High levels of bio-ADM are positively associated with ARDS in a general ICU population, whereas both high and low levels of bio-ADM are associated with mortality in ARDS, possibly due to the dual action of bio-ADM in stabilising the endothelium and causing vasodilation. Further studies are needed to elucidate the role of bio-ADM in ARDS development and studies targeting bio-ADM for treatment and possibly prevention of ARDS.

## Supplementary Information

**Additional file 1.** **Table S1. Characteristics of control population and patients fulfilling ARDS critera but lacking radiology**. Data regarding general characteristics, outcomes, organ dysfunction and illness severity are presented below. Patients included in the study were compared to those excluded, and the p-values refer to that comparison.Proportions (%) are within their subgroups unless otherwise specified. SD: standard deviation; IQR: interquartile range; CCI: Charlson Comorbidity Index; SAPS3: Simplified Acute Physiology Score III; SOFA: Sequential Organ Failure Assessment; bio-ADM: circulating bioactive adrenomedullin; CRP: C-Reactive Protein; ICU: intensive care unit. IMV: invasive mechanical ventilation. **Table S2. Characteristics of included and excluded patients.** Data regarding general characteristics, outcomes, organ dysfunction and illness severity are presented below. Patients included in the study were compared to those excluded, and the p-values refer to that comparison.Proportions (%) are within their subgroups unless otherwise specified. SD: standard deviation; IQR: interquartile range; CCI: Charlson Comorbidity Index; SAPS3: Simplified Acute Physiology Score III; SOFA: Sequential Organ Failure Assessment; bio-ADM: circulating bioactive adrenomedullin; CRP: C-Reactive Protein; ICU: intensive care unit. IMV: invasive mechanical ventilation.

## Data Availability

The datasets generated and analysed during the current study are not publicly available due to limitations in the ethical approval of the study and data management policies of Region Skåne. However, they are available from the corresponding author upon reasonable request.

## Abbreviations

ADM: Adrenomedullin
ARDS: Acute respiratory distress syndrome
AUC: Area under the curve
bio-ADM: Circulating bioactive adrenomedullin
CI: Confidence interval
CRP: C-reactive protein
CRRT: Continuous renal replacement therapy
CT: Computed tomography
ICU: Intensive care unit
IQR: Interquartile range
N/A: Not applicable
OR: Odds ratio
ROC: Receiver operating characteristic
SAPS3: Simplified acute physiology score III
SIR: Swedish intensive care registry
SOFA: Sequential organ failure assessment
