# Supplementary material for: Plasma bioactive adrenomedullin on intensive care unit admission is associated with acute respiratory distress syndrome: an observational study
Source: Intensive Care Med Exp. 2023 Mar 3;11:10. doi: 10.1186/s40635-023-00494-7 (PMC9981258; doi:10.1186/s40635-023-00494-7)
Supplement: Supplementary file 1 — Additional file 1. Table S1. Characteristics of control population and patients fulfilling ARDS critera but lacking radiology. Data regarding general characteristics, outcomes, organ dysfunction and illness severity are presented below. Patients included in the study were compared to those excluded, and the p-values refer to that comparison.Proportions (%) are within their subgroups unless otherwise specified. SD: standard deviation; IQR: interquartile range; CCI: Charlson Comorbidity Index; SAPS3: Simplified Acute Physiology Score III; SOFA: Sequential Organ Failure Assessment; bio-ADM: circulating bioactive adrenomedullin; CRP: C-Reactive Protein; ICU: intensive care unit. IMV: invasive mechanical ventilation. Table S2. Characteristics of included and excluded patients. Data regarding general characteristics, outcomes, organ dysfunction and illness severity are presented below. Patients included in the study were compared to those excluded, and the p-values refer to that comparison.Proportions (%) are within their subgroups unless otherwise specified. SD: standard deviation; IQR: interquartile range; CCI: Charlson Comorbidity Index; SAPS3: Simplified Acute Physiology Score III; SOFA: Sequential Organ Failure Assessment; bio-ADM: circulating bioactive adrenomedullin; CRP: C-Reactive Protein; ICU: intensive care unit. IMV: invasive mechanical ventilation. [file 40635_2023_494_MOESM1_ESM.pdf]

Supplementary table 1. ARDS patients vs patients who fulfilled ARDS criteria but lacked chest radiology

Parameter	ARDS without radiology	ARDS	P-value	Missing (%)
**Demographics**				
n	36	132		
Female, n (%)	9 (25.0)	43 (32.6)	0.504	
Age, years, mean (SD)	63.08 (14.17)	64.04 (14.28)	0.722	
BMI, mean (SD)	26.67 (8.04)	26.48 (6.25)	0.063	35.7
**Comorbidities**				
CCI score, mean (SD)	3.44 (2.40)	3.62 (2.40)	0.695	
Cardiac arrest, n (%)	1 (2.8)	4 (3.0)	1.000	
Sepsis-3 criteria, n (%)	17 (47.2)	89 (67.4)	0.042	
**Direct lung-injury risk factors, n (%)**				
Pneumonia	14 (38.9)	95 (72.0)	<0.001	
Aspiration	8 (22.2)	32 (24.2)	0.975	
Inhalational injury	1 (2.8)	0 (0.0)	0.485	
Drowning	0 (0.0)	0 (0.0)	NA	
Lung contusion	0 (0.0)	4 (3.0)	0.660	
Pulmonary vasculitis	0 (0.0)	4 (3.0)	0.660	
Other	0 (0.0)	2 (1.5)	1.000	
None	14 (38.9)	15 (11.4)	<0.001	
**Indirect lung-injury risk factor, n (%)**				
Extrapulmonary sepsis	12 (33.3)	38 (28.8)	0.747	
Severe burns	0 (0.0)	0(0.0)	NA	
Non-cardiogenic shock	4 (11.1)	7 (5.3)	0.385	
Drug overdose	1 (2.8)	7 (5.3)	0.850	
Multiple blood transfusions	7 (19.4)	9 (6.8)	0.049	
Trauma	1 (2.8)	2 (1.5)	1.000	
Pancreatitis	0 (0.0)	1 (0.8)	1.000	
Other	0 (0.0)	0 (0.0)	NA	
None	16 (44.4)	76 (57.6)	0.225	
**Severity of illness, mean (SD)**				
SAPS 3 score	66.86 (17.90)	72.24 (13.98)	0.056	
SOFA score, total	9.26 (4.09)	9.75 (3.75)	0.517	8.9
Respiratory SOFA	3.03 (0.71)	3.29 (0.73)	0.060	7.7
Coagulation SOFA	0.48 (0.83)	0.51 (0.93)	0.878	8.3
Neurologic SOFA	1.72 (1.61)	1.63 (1.53)	0.761	0.6
Renal SOFA	1.26 (1.63)	1.41 (1.51)	0.594	6.0
Circulatory SOFA	2.54 (1.63)	2.52 (1.59)	0.947	0.6
Hepatic SOFA	0.36 (0.78)	0.42 (0.89)	0.741	9.5
**Biochemistry on admission**				
Bio-ADM, pg/L median [IQR]	38.61 [21.25, 78.39]	43.04 [22.00, 96.52]	0.555	
CRP, mg/L median [IQR]	47.00 [9.57, 130.50]	99.00 [32.50, 229.75]	0.010	1.2
Leukocytes, 10^9/L, median [IQR]	14.40 [9.90, 20.38]	13.95 [9.17, 18.85]	0.416	1.2
Lactate, mmol/L median [IQR]	4.00 [2.10, 5.90]	3.10 [1.80, 5.20]	0.181	0.6
Creatinine, umol/L, median [IQR]	102.50 [79.75, 149.00]	120.00 [84.00, 188.50]	0.273	0.6
PF-ratio day 1	23.96 (8.63)	21.06 (9.20)	0.092	1.2
**Outcomes**				
Mortality, n (%)	11 (30.6)	52 (39.4)	0.437	
Duration of ICU stay, d, median [IQR]	2.08 [0.84, 4.64]	3.49 [1.74, 6.46]	0.010	
Duration of IMV, d, median [IQR]	1.25 [0.46, 2.97]	2.26 [0.29, 4.13]	0.524	

Supplementary table 2. Included vs excluded patients

Parameter	INCLUDED	EXCLUDED	P-value	Missing (%)
**Demographics**				
n	1224	367		
Female, n (%)	472 (38.6)	136 (37.1)	0.646	
Age, years, mean (SD)	62.03 (16.97)	57.30 (18.75)	<0.001	
BMI, mean (SD)	26.87 (6.62)	26.37 (6.35)	0.284	30.0
**Comorbidities**				
CCI score, mean (SD)	3.62 (2.73)	3.05 (2.76)	<0.001	
Cardiac arrest, n (%)	15 (1.2)	14 (3.8)	0.002	0.4
**Direct lung-injury risk factors, n (%)**				
Pneumonia	227 (18.5)	80 (21.8)	0.190	
Aspiration	103 (8.4)	38 (10.4)	0.297	
Inhalational injury	3 (0.2)	2 (0.5)	0.712	
Drowning	2 (0.2)	0 (0.0)	1.000	
Lung contusion	27 (2.2)	18 (4.9)	0.011	
Pulmonary vasculitis	7 (0.6)	2 (0.5)	1.000	
Other	3 (0.2)	1 (0.3)	1.000	
None	902 (73.7)	252 (68.7)	0.068	
**Indirect lung-injury risk factor, n (%)**				
Extrapulmonary sepsis	201 (16.4)	49 (13.4)	0.182	
Severe burns	1 (0.1)	2 (0.5)	0.268	
Non-cardiogenic shock	99 (8.1)	39 (10.6)	0.159	
Drug overdose	66 (5.4)	29 (7.9)	0.098	
Multiple blood transfusions	115 (9.4)	39 (10.6)	0.549	
Trauma	96 (7.8)	41 (11.2)	0.059	
Pancreatitis	11 (0.9)	4 (1.1)	0.980	
Other	30 (2.5)	6 (1.6)	0.470	
None	721 (58.9)	196 (53.4)	0.070	
**Severity of illness, mean (SD)**				
SAPS 3 score	59.11 (16.54)	57.11 (16.19)	0.041	0.1
SOFA score, total	6.45 (3.82)	6.56 (3.67)	0.676	15.7
Respiratory SOFA	2.09 (1.28)	2.01 (1.22)	0.426	27.1
Coagulation SOFA	0.32 (0.71)	0.29 (0.68)	0.536	19.5
Neurologic SOFA	1.29 (1.53)	1.51 (1.64)	0.052	9.9
Renal SOFA	0.90 (1.37)	0.85 (1.34)	0.654	18.5
Circulatory SOFA	1.79 (1.66)	1.63 (1.56)	0.179	9.9
Hepatic SOFA	0.28 (0.64)	0.25 (0.60)	0.611	21.7
**Biochemistry on admission**				
Bio-ADM, pg/L median [IQR]	38.61 [21.26, 78.94]	-		
CRP, mg/L median [IQR]	26.00 [4.32, 118.00]	36.00 [5.18, 133.25]	0.404	3.8
Leukocytes, 10^9/L, median [IQR]	13.30 [9.20, 18.10]	12.45 [9.10, 17.17]	0.388	3.1
Lactate, mmol/L median [IQR]	2.40 [1.40, 4.43]	1.90 [1.20, 3.70]	0.001	6.0
Creatinine, median [IQR]	95.00 [72.00, 141.00]	96.00 [69.00, 138.00]	0.266	2.0
**ARDS-severity, n (%)**				
ARDS, total n	132 (10.8)	21 (5.7)	0.005	0.0
Mild ARDS	24 (18.2)	7 (33.3)		
Moderate ARDS	81 (61.4)	12 (57.1)		
Severe ARDS	27 (20.5)	2 (9.5)		
**Outcomes**				
Mortality, n (%)	246 (20.1)	116 (31.7)	<0.001	0.1
Duration of ICU stay, d, median [IQR]	1.57 [0.78, 3.32]	1.49 [0.63, 2.87]	0.123	0.1
Duration of IMV, d, median [IQR]	1.25 [0.46, 3.05]	1.24 [0.44, 2.81]	0.396	
